# Structure and Magnetism of Mn_5_Ge_3_ Nanoparticles

**DOI:** 10.3390/nano8040241

**Published:** 2018-04-15

**Authors:** Onur Tosun, Mohammed Salehi-Fashami, Balamurugan Balasubramanian, Ralph Skomski, David J. Sellmyer, George C. Hadjipanayis

**Affiliations:** 1 Department of Physics and Astronomy, University of Delaware, Newark, DE 19711, USA; onurt@udel.edu (O.T.); mfashami@udel.edu (M.S.-F.); 2 Department of Physics and Astronomy, University of Nebraska, Lincoln, NE 68588, USA; balamurugan@unl.edu (B.B.); rskomski@neb.rr.com (R.S.); dsellmyer@unl.edu (D.J.S.); 3 Nebraska Center for Materials and Nanoscience, University of Nebraska, Lincoln, NE 68588, USA

**Keywords:** magnetic nanoparticles, cluster deposition, magnetization

## Abstract

In this work, we investigated the magnetic and structural properties of isolated Mn_5_Ge_3_ nanoparticles prepared by the cluster-beam deposition technique. Particles with sizes between 7.2 and 12.6 nm were produced by varying the argon pressure and power in the cluster gun. X-ray diffraction (XRD)and selected area diffraction (SAD) measurements show that the nanoparticles crystallize in the hexagonal Mn_5_Si_3_-type crystal structure, which is also the structure of bulk Mn_5_Ge_3_. The temperature dependence of the magnetization shows that the as-made particles are ferromagnetic at room temperature and have slightly different Curie temperatures. Hysteresis-loop measurements show that the saturation magnetization of the nanoparticles increases significantly with particle size, varying from 31 kA/m to 172 kA/m when the particle size increases from 7.2 to 12.6 nm. The magnetocrystalline anisotropy constant *K* at 50 K, determined by fitting the high-field magnetization data to the law of approach to saturation, also increases with particle size, from 0.4 × 10^5^ J/m^3^ to 2.9 × 10^5^ J/m^3^ for the respective sizes. This trend is mirrored by the coercivity at 50 K, which increases from 0.04 T to 0.13 T. A possible explanation for the magnetization trend is a radial Ge concentration gradient.

## 1. Introduction

Nanostructuring often adds functionality to bulk materials and yields new and interesting physics. This includes nanoparticles where size and surface effects are both scientifically important and attractive for a variety of advanced technologies [1,2,3,4,5,6]. Mn-based intermetallic compounds are being investigated as potential new rare-earth-metal-free permanent magnets and as magnetic materials with interesting spin-electronic properties [7,8]. In particular, Mn_5_Ge_3_ compound is a promising candidate for spin-based-device applications due to its high spin polarization and injection efficiency within semiconductor matrices [9,10,11,12,13], its thermal stability, and the possibility to improve its magnetic properties through carbon, iron, and antimony substitutions [14,15,16,17,18,19]. 

Bulk Mn_5_Ge_3_ is a ferromagnetic intermetallic compound characterized by a Curie temperature *T*_c_ near room temperature and an easy magnetization direction along the *c* axis of the hexagonal crystal structure [20]. It crystallizes in the hexagonal D8_8_ structure (prototype Mn_5_Si_3_ and space group P6_3_/mcm) and has lattice parameters of *a* = 7.184 Å and *c* = 5.053 Å. The unit cell contains 10 Mn atoms, which crystallographically occupy very different 4d (Mn1) and 6h (Mn2) sites [21]. The saturation magnetization *M*_s_ of the compound is nearly entirely provided by the Mn atoms, and the experimental bulk saturation magnetization is about 2.60 *μ*_B_ at 4.2 K [22] which agrees well with the density functional theory (DFT) prediction of 2.5 *μ*_B_ by S. Picozzi et al. [9]. The lowest-order anisotropy constant was determined experimentally by Y. Tawara et al., who found *K* = 4.2 × 10^5^ J/m^3^ [20].

There are several reports on the magnetic and structural properties of Mn_5_Ge_3_ in the form of thin films [23,24], nanoislands [25], and nanomagnets embedded in semiconductor matrices by ion implantation and molecular beam epitaxy [26,27,28,29,30]. It has been reported that, regardless of the fabrication method used, precipitates of small Mn_5_Ge_3_ nanoclusters may occur due to the high growth temperature as well as the Mn content exceeding the low solubility in Ge [31,32,33]. Therefore, the fabrication of isolated Mn_5_Ge_3_ nanoparticles by cluster-beam deposition (CBD) is very important in terms of being able to prepare the particles with a single-step deposition process without the need for any heat-treatment and with control over the phase purity and crystallinity [34,35]. Moreover, free nanoparticles can be used as prototype models for the nanoclusters formed in semiconductor matrices, boundaries, and surfaces of thin films, as well as for the understanding of size effects on their structural and magnetic properties. 

In this paper, we describe the synthesis of isolated Mn_5_Ge_3_ nanoparticles, which have not yet been investigated. Moreover, we report their basic magnetic properties and investigate the effect of particle size and temperature on the magnetism of the particles. 

## 2. Materials and Methods 

**Figure 1:**
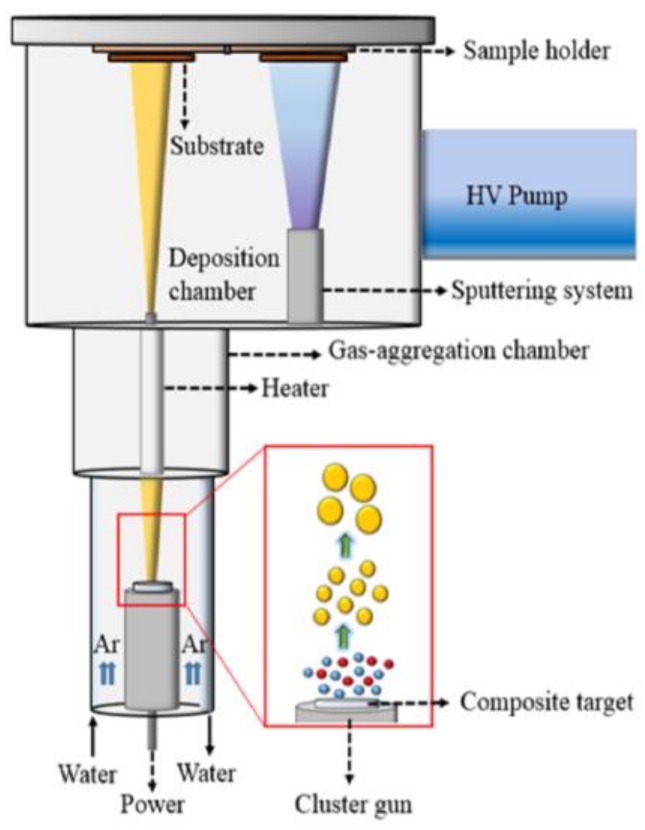
Fabrication of Mn_5_Ge_3_ nanoparticles. A schematic illustration of the cluster-beam-deposition (CBD) process.

Three samples of Mn_5_Ge_3_ nanoparticles having average sizes of 7.2 nm (S1), 10 nm (S2), and 12.6 nm (S3) were fabricated using the CBD method as schematically shown in Figure 1. The samples were produced by sputtering a compacted Mn_5_Ge_3_ powder target, which was prepared by arc-melting high purity elements (99.9% for Mn and 99.9% for Ge), using a high DC power of 60–80 W under a high-purity Ar (99.9999%) pressure of 1.0, 1.3, and 1.6 Torr for the samples S1, S2, and S3, respectively. The nanoparticles formed travel through a narrow orifice and are deposited on 500-μm thick Si wafer and on C-coated Cu grids in the deposition chamber for magnetic and microstructure measurements, respectively. A silver capping layer of 13 nm was deposited by magnetron sputtering at a DC power of 5 W in order to prevent the oxidation of the nanoparticulate film samples by exposure to air. No in-flight heat treatment was conducted. Magnetic contributions due to the Si wafer, Ag coat, and Kapton tape were properly determined and subtracted from the raw data. The compositional analysis was performed with a JEOL JSM 6330F Scanning Electron Microscope (SEM) (JEOL Ltd., Akishima, Tokyo, Japan). For the structural analysis of the Mn_5_Ge_3_ nanoparticles we used a Rigaku Ultima IV X-ray diffractometer (Rigaku Corp., Akishima, Tokyo, Japan) using Cu Kα radiation and operating with a wavelength of 1.540 Å, as well as a JEOL JEM-3010 Transmission Electron Microscope (TEM) (JEOL Ltd., Akishima, Tokyo, Japan) operating at 300 kV. The magnetic properties of the nanoparticles were carried out with a Quantum Design Versa Lab Vibrating Sample Magnetometer (VSM) (Quantum Design Inc., San Diego, CA, US).

## 3. Results and Discussion

### 3.1. Crystal Structure and Microstructure Measurements

**Figure 2:**
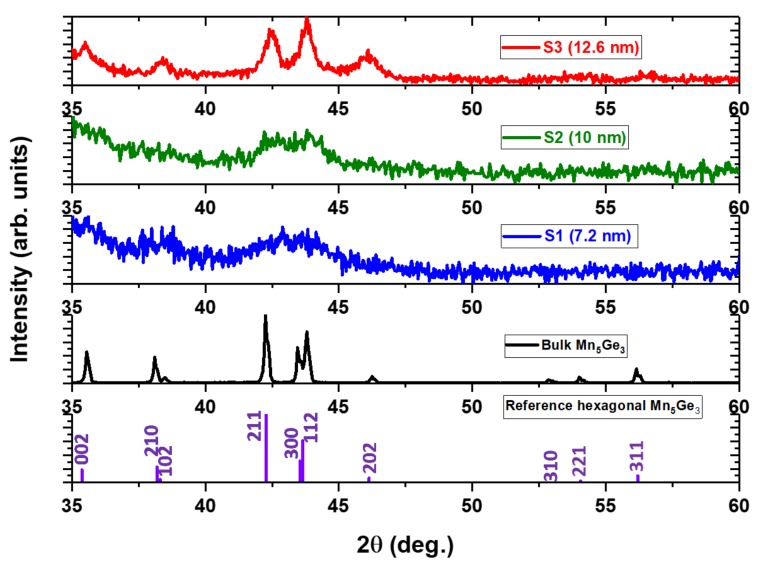
X-ray diffraction (XRD) patterns for the reference Mn_5_Si_3_-type hexagonal crystal structure, for the bulk Mn_5_Ge_3_ and for the samples S1, S2, and S3.

The stoichiometry of the samples was determined by energy-dispersive spectroscopy (EDS); the measurements showed that all of our Mn_5_Ge_3_ nanoparticles had a composition similar to that of the sputtering target (Mn_62.5_Ge_37.5_). Figure 2 compares the x-ray diffraction (XRD) patterns of the samples S1, S2, and S3 with that of Mn_5_Ge_3_ powder made from the sputtering target and with the standard diffraction data of hexagonal bulk Mn_5_Ge_3_. The broad XRD peak of sample S1 in Figure 2 indicates that the Mn_5_Ge_3_ nanoparticles are small and disordered. The split in the broad XRD peak observed in sample S2 is due to the increase in both the particle size and crystallinity order. All of the XRD reflections of the hexagonal structure become visible as the nanoparticle size increases, as can be seen in the XRD data of sample S3.

**Figure 3:**
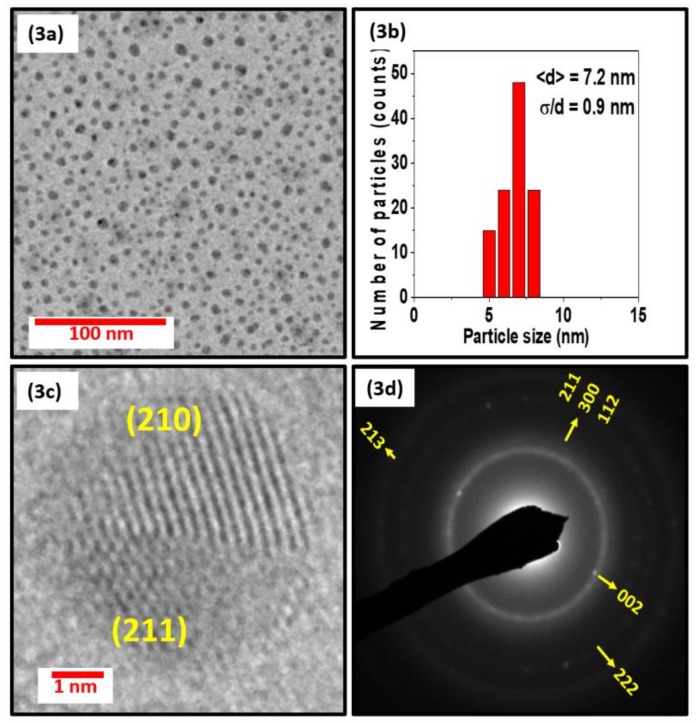
Figure (**3a**), (**3b**), (**3c**), and (**3d**) show the particles by TEM, the size distribution, HRTEM, and selected area diffraction (SAD) images of the nanoparticles of sample S1, respectively.

**Figure 4:**
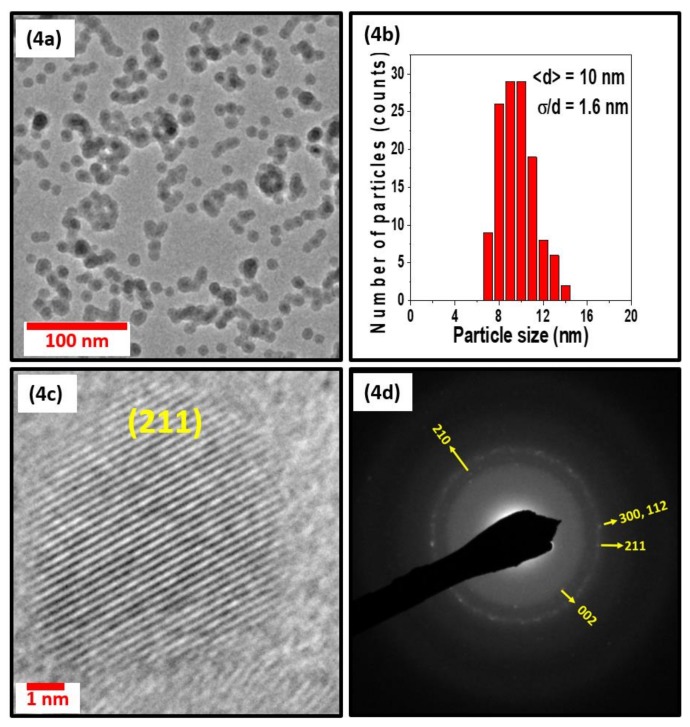
Figure (**4a**), (**4b**), (**4c**), and (**4d**)) show the particles observed by TEM, the size distribution, HRTEM, and SAD images of the nanoparticles of sample S2, respectively.

**Figure 5:**
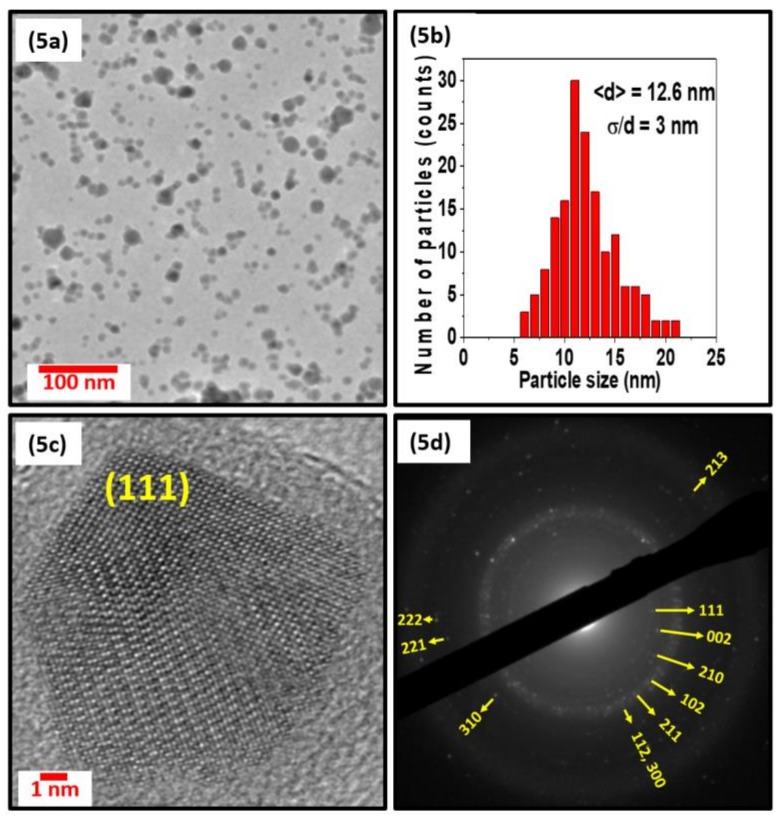
Figure (**5a**), (**5b**), (**5c**), and (**5d**) show the particles observed by TEM, the particle size distribution, HRTEM, and SAD images of the nanoparticles of sample S3, respectively.

Figure 3, Figure 4 and Figure 5 show the TEM, size distribution, high resolution TEM (HRTEM), and selected area diffraction (SAD) images of the samples S1, S2, and S3. The SAD rings for the nanoparticles with different sizes in Figure 3d, Figure 4d, and Figure 5d are indexed to the Mn_5_Si_3_-type hexagonal crystal structure with space group P6_3_/mcm. The difference in brightness of the SAD rings can be attributed to the size difference of the particles, which can also be deduced from the XRD data in Figure 2. The respective TEM images in Figure 3a, Figure 4a, and Figure 5a show uniformly distributed nanoparticles with average sizes of 7.2 nm, 10 nm, and 12.6 nm, along with the corresponding particle size distributions in Figure 3b, Figure 4b, and Figure 5b. The selected area electron diffraction and HRTEM images are consistent with the XRD data, showing d-spacing values that can be fitted to the hexagonal crystal structure, as shown in Figure 3c, Figure 4c, and Figure 5c. It is interesting to note that some of the nanoparticles are polycrystalline despite their small size. The nanoparticles have the same crystal structure as the bulk sample, although their lattice parameters are slightly different. Lattice parameters were determined by fitting the XRD data to the reference data obtained by Y. Zhang et al. [36] using the software reported in Reference [37] and are presented in Table 1.

### 3.2. Magnetic Properties

**Figure 6:**
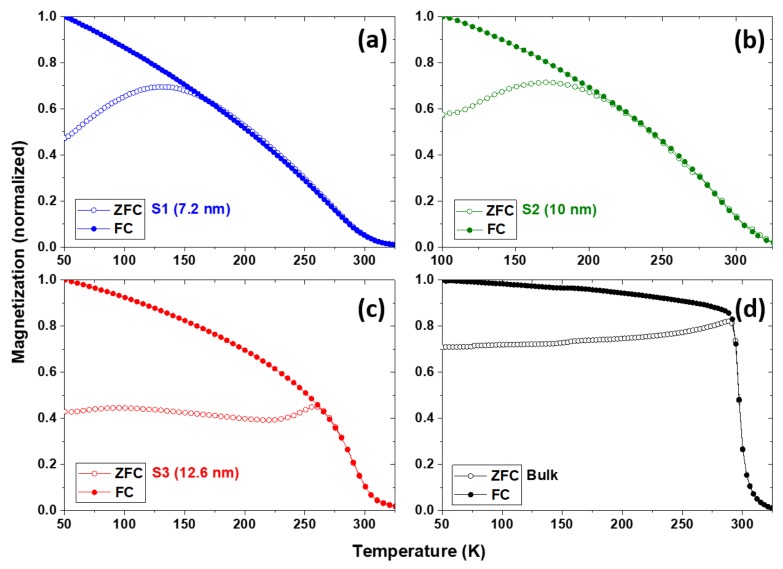
Temperature-dependent magnetization curves of the samples (**a**) S1; (**b**) S2; (**c**) S3; and (**d**) powdered bulk measured at 0.05 T.

The temperature dependence of the magnetic properties of the nanoparticles was measured by the vibrating sample magnetometer(VSM) and is shown in Figure 6. Figure 6a–c show the zero-field-cooled (ZFC) and field-cooled (FC) magnetization curves at 0.05 T for S1, S2, and S3, respectively. Since the nanoparticles have fairly broad particle size distributions, as shown in the graphs of Figure 3b, Figure 4b, and Figure 5b, it is difficult to distinguish whether the broad maxima in the M vs. T curve are related to the blocking temperature or the Hopkinson effect. As the particle size increases, the temperature at which the ZFC curves exhibit broad maxima increase but the Curie temperatures do not change much with size. It was also found that the decrease in magnetization with increasing temperature becomes steeper as the particle size and crystallinity increase.

**Figure 7:**
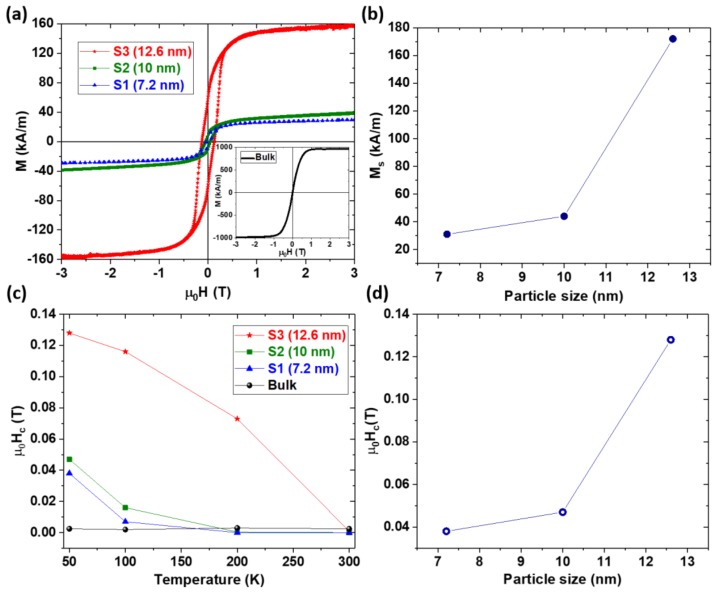
Magnetization and extrinsic magnetic properties of the nanoparticles: (**a**) hysteresis loops measured at 50 K; (**b**) saturation magnetization versus particle size; (**c**) coercivity versus temperature; and (**d**) coercivity versus particle size.

Figure 7a shows the hysteresis loops of all of the samples measured at 50 K. The saturation magnetization of the nanoparticles increases dramatically with particle size. The saturation magnetization of the bulk sample at 50 K is *M*_S_ = 972 kA/m, in S3 it is *M*_S_ = 172 kA/m, in S2 it is *M*_S_ = 44 kA/m, and in S1 it is *M*_S_ = 31 kA/m. The corresponding average magnetic moments per Mn atom are 2.35 *μ*_B_, 0.420 *μ*_B_, 0.110 *μ*_B_, and 0.075 *μ*_B_, respectively. The magnetocrystalline anisotropy constant *K* was estimated by fitting the high field *M*(*H*) data obtained at 50 K to the law of approach to saturation using the expression M = M_S_(1 − A/H^2^) + χH for uniaxial isotropic particles [38,39], where A = (4/15)(K_1_^2^/M_S_^2^) and χ is the high-field susceptibility. This analysis yields the values of *K* determined for the bulk, S1, S2, and S3 samples as 4 × 10^5^ J/m^3^, 0.4 × 10^5^ J/m^3^, 0.9 × 10^5^ J/m^3^, and 2.9 × 10^5^ J/m^3^, respectively. The coercivity *H*_c_ of samples S1 and S2 decrease dramatically with increasing temperature while the coercivity of sample S3 of relatively larger particles decreases in a different manner than in that in S1 and S2. 

A striking feature of the present system is the pronounced magnetization reduction with decreasing particle size, from 172 kA/m for 12.7 nm to 31 kA/m for 7.2 nm. There are several possible explanations for the low magnetization, such as a collapse of the magnetic moment per atom, different ferrimagnetic spin states, noncollinear magnetism, or surface magnetism. Several structural features can trigger these magnetic changes, for example particle-size-dependent off-stoichiometry, radial Ge concentration gradients, solubility range with respect to neighboring nanoparticle phases, defect densities, and crystallographic indexing of the surfaces. Minor structural changes are known to sometimes have a great effect on the magnetism of elements in the middle of the transition-metal series. For example, the Curie temperature of Fe_5_Si_3_ increases by a factor 1/2 if the Fe concentration increases from 62.5 at % to 65.0 at % [40]. From a basic viewpoint, there are two reasons for this behavior. First, the systems in question are close to the onset of magnetic order or close to transitions between different types of orders. Second, elements in the middle of the iron series, such as Mn, tend to form antiferromagnetic bonds, which leads to a pronounced competition between ferromagnetic, ferrimagnetic, and noncollinear spin states.

At this stage, it is difficult to say whether the particle size reduction yields any minor structural changes that strongly reduce the net magnetization in the particle cores. However, such a magnetization reduction inside the particles is not the only possible explanation. The magnetization in the nanoparticles may be inhomogeneous, for example due to different neighborhoods and exchange bonds *J*_ij_ at the particle surface, or due to a processing-related radial Ge concentration gradient. 

Let us consider, for simplicity, a semi-phenomenological model where the core and shell regions have different magnetizations, denoted as *M*_c_ and M*, respectively. For a shell of thickness Δ, the corresponding net magnetization is:

*M*_s_ = (1 − *f*) *M*_c_ + f M*, (1)

where *f* = 1 − (*D* − 2Δ)^3^/D^3^ is the volume fraction of the shell. Applying Equation (1) to two particle sizes *D* (7.2 nm and 12.7 nm) yields two coupled linear equations with two unknowns (*M*_c_ and M*), slightly complicated by the nonlinear character of *f*. The solution of the two equations depends on Δ and therefore on the underlying physical mechanism. Since there is no notable change in the Curie temperatures of the particles, it is fair to assume that the cores of the particles have magnetic properties similar to the bulk. If we assume that a Ge concentration gradient or some other structural feature yields a nonmagnetic shell (M* = 0) of thickness Δ, and take the magnetization of the core to be identical to that of the bulk (972 kA/m), then a 12.6-nm nanoparticle with a dead layer having a thickness of about 2.8 nm reproduces the saturation magnetizations of both the 7.2-nm and 12.6-nm particles. In other words, a single parameter (Δ) semi-quantitatively explains the net magnetization across the whole particle size range between 7.2 nm and 12.6 nm. 

## 4. Conclusions

In summary, we used cluster-beam deposition to prepare isolated Mn_5_Ge_3_ nanoparticles with sizes in the range of 7.2 nm to 12.6 nm. The saturation magnetization and magnetocrystalline anisotropy increase significantly with particle size, from *M*_s_ = 31 kA/m to 172 kA/m and from *K* = 0.4 × 10^5^ J/m^3^ to 2.9 × 10^5^ J/m^3^ as the particle size increases. In contrast, the Curie temperature does not vary much with size. Because of their relatively high anisotropy, all of the particles exhibit low-temperature coercivities varying from 0.04 T in the smallest particles to 0.13 T in the largest particles, compared to the very small (nearly zero) coercivity of the bulk sample. A tentative explanation of the observed magnetization increase with particle size is provided by a simple core-shell model with a 2.8-nm shell having zero magnetization. A detailed description of the magnetization and anisotropy trends as a function of particle size remains a challenge for future research.

## Figures and Tables

**Table 1:** Average particle sizes obtained by TEM measurements, using Scherrer’s equation, and lattice parameters obtained by fitting the XRD peaks in Figure 2.

Average Particle Size (nm) (TEM)	Average Particle Size (nm) (Scherrer’s Equation)	Lattice Parameters (Å) (Fitting)
S1 7.2	7.3	*a* = *b* = 7.183 Å, *c* = 5.080 Å
S2 10	12.2	*a* = *b* = 7.167 Å, *c* = 5.011 Å
S3 12.6	16.7	*a* = *b* = 7.193 Å, *c* = 5.078 Å
